# Amyloid‐Related Imaging Abnormalities, ARIAs: Experience of a Single Center in the Context of Clinical Trials with Anti‐Amyloid Antibodies

**DOI:** 10.1002/alz70859_102269

**Published:** 2025-12-25

**Authors:** Ainhoa Atorrasagasti Villar, Rafael Angel Villino‐Rodríguez, Christian Espinoza‐Vinces, Cristina Pérez‐Prol, Adolfo Jimenez‐Huete, Pablo Dominguez, Mario Riverol

**Affiliations:** ^1^ Clinica Universidad de Navarra, SAN SEBASTIAN, Navarra Spain; ^2^ University Clinica of Navarra, Pamplona, Navarra Spain; ^3^ University Clinic of Navarra, Pamplona, Navarra Spain; ^4^ University Clinic of Navarra, Pamplona, Navarra Spain

## Abstract

**Background:**

Amyloid‐related imaging abnormalities (ARIAs) are complications associated with anti‐amyloid therapy in Alzheimer’s disease (AD). ARIAs include ARIA‐edema (ARIA‐E) and ARIA‐hemorrhage (ARIA‐H), which may present asymptomatically or with mild symptoms. Identifying risk factors, such as the ApoE4 genotype, and understanding the clinical course of ARIAs are critical for optimizing treatment outcomes in AD patients undergoing anti‐amyloid therapy.

**Method:**

A retrospective observational analysis was conducted on data from patients participating in clinical trials involving anti‐amyloid antibodies (Aducanumab, Gantenerumab, Crenezumab). Demographic data, ApoE genotyping, cardiovascular risk factors, treatment initiation dates, and occurrence of ARIAs were recorded. ARIA events were classified as ARIA‐E or ARIA‐H, and associated symptoms, time of onset, and resolution were documented. Brain MRI was used to monitor ARIA progression and persistence.

**Result:**

The study included 52 patients (mean age: 68.6 years; range: 52–85), 42.3% of whom were female. ARIAs were identified in 9 patients, accounting for 15 events: 7 treated with Aducanumab and 2 with Gantenerumab, while none occurred with Crenezumab. Among these, 5 cases involved ARIA‐E, 9 involved ARIA‐H, and 1 patient exhibited both. Most events were asymptomatic or associated with very mild symptoms, with no treatment discontinuation required. ARIA‐E typically resolved spontaneously within 4–8 weeks, while ARIA‐H persisted on follow‐up MRIs. Events generally occurred between weeks 20 and 39 of treatment, with ARIA‐H appearing later in some exceptional cases. A strong correlation with the ApoE4 genotype was observed, as most patients developing ARIAs carried at least one ApoE4 allele.

**Conclusion:**

Patients undergoing anti‐amyloid therapy are at risk of developing ARIAs, particularly those carrying the ApoE4 genotype. ARIAs are generally asymptomatic, do not necessitate treatment discontinuation, and show differing courses depending on the subtype: ARIA‐E resolves spontaneously, while ARIA‐H persists. Aducanumab was associated with the highest frequency of ARIA events. These findings underscore the importance of regular MRI monitoring and patient‐specific management strategies to mitigate complications and optimize therapy outcomes.

